# The Impact of the COVID-19 Epidemic on the Mood and Diet of Patients Undergoing Bariatric Surgery

**DOI:** 10.3390/nu14142849

**Published:** 2022-07-12

**Authors:** Iwona Boniecka, Aneta Czerwonogrodzka-Senczyna, Marzena Sekuła, Piotr Zawodny, Marcin Szemitko, Magdalena Sieńko, Jerzy Sieńko

**Affiliations:** 1 Department of Clinical Dietetics, Faculty of Health Sciences, Medical University of Warsaw, E Ciołka Str. 27, 01-445 Warsaw, Poland; aneta.czerwonogrodzka@wum.edu.pl; 2 Interdisciplinary Doctoral School of Social Sciences and Humanities, SWPS University of Social Sciences and Humanities, Chodakowska Str. 19/31, 03-815 Warsaw, Poland; msekula1@st.swps.edu.pl; 3 Zawodny Clinic, Ku Słońcu Str. 58, 71-047 Szczecin, Poland; estetic@estetic.pl; 4 Department of Interventional Radiology, Pomeranian Medical University, Al. Pow. Wielkopolskich Str. 72, 70–111 Szczecin, Poland; szemitko@gmail.com; 5 Department of Pediatrics, Endocrinology, Diabetology, Metabolic Diseases and Cardiology, Pomeranian Medical University in Szczecin, Unii Lubelskiej Str. 1, 71-252 Szczecin, Poland; brzoskomag@poczta.onet.pl; 6 Department of General Surgery and Transpalntation, Pomeranian Medical University, Al. Pow. Wielkopolskich Str. 72, 70–111 Szczecin, Poland; jsien@poczta.onet.pl

**Keywords:** bariatric surgery, diet, mood, snacking, COVID-19, pandemic

## Abstract

Limited social contacts, lack of professional activities, economic insecurity, and a sense of threat, as well as boredom during the COVID-19 pandemic, contributed to tension and stress. All of these increase the risk of an inappropriate diet. The aim of this cross-sectional study was to assess the impact of the COVID-19 pandemic on the mood and nutrition of patients undergoing bariatric surgery. A group of 312 patients (both before and after bariatric surgery) completed a questionnaire about their diet and mood during COVID-19 lockdown. About 70% of all respondents reacted to the epidemiological situation: irritability, anxiety about their own health, and eating without being hungry. A total of 74% of all of the subjects snacked between meals (especially sweets). The respondents who believed that obesity and its complications had a negative impact on the prognosis of the coronavirus infection had a statistically significant higher prevalence of health anxiety, feeling that important life issues were out of control, irritability, need for psychological support, and need for dietary consultation. Patients after bariatric surgery had e.g., a statistically significant lower incidence of feeling hungry, eating after meals, and eating fatty foods. The COVID-19 pandemic has been shown to negatively affect the mood and diet of bariatric patients, which may affect their health status and worsen the prognosis of COVID-19.

## 1. Introduction

On 31 December 2019, the WHO was informed of cases of pneumonia of an unknown cause in Wuhan City, China. A novel coronavirus was identified in early January 2020 and it was named the “COVID-19 virus”. On 11 March 2020, a rapid increase in the cases outside China prompted the WHO Director-General to announce a pandemic state [1,2]. It forced an unprecedented scale of isolation, a limitation of social and professional contacts and a reduction in physical movement. Schools, public and cultural institutions, and sports facilities were closed, which resulted in a significant limitation on physical activity and had a negative impact on the health and mental well-being of society [3,4]. Access to healthcare declined, many elective surgeries (including bariatric surgeries) were canceled, and hospitals and hospital wards were converted to treat this spreading infection [3,4,5]. Patients with obesity who were both in the process of preparation (several months) for surgery and after surgery often had limited contact with a dietitian, surgeon, or psychologist, which, according to Polish standards, should be provided [6]. Limited social contacts, a pause on professional activity, economic insecurity, and a sense of threat, as well as the daily routine and boredom, contributed to the tension and stress. Boredom, in turn, is associated with a greater consumption of foods rich in carbohydrates and energy [7,8]. Moreover, during a quarantine period, constantly listening to or reading information about a pandemic can exacerbate stress [8]. Chronic stress often leads to overeating, mainly with sweet snacks and reaching for so-called “comfort foods” [7,8,9,10]. This desire to consume a particular type of food is defined as a “food craving”, and is a concept that includes emotional (intense craving), behavioral (searching for food), cognitive (thoughts about eating), and physiological (drooling) processes [11]. Dopamine, produced upon feeling satisfied after eating, activates the joy sites in the brain. This motivates a person to eat certain foods to enjoy this feeling of contentment or to alleviate negative feelings [9,10]. A review by Adam et al. describes the mediator role of the reward system in the relationship between stress and stress-induced eating [12]. The hypothalamic–pituitary–adrenal (HPA) axis plays a key role in this process. The adequate regulation of energy and food intake under stress is important for survival. Severe stress can result in higher cortisol levels, leading to an activation of the HPA axis, which interacts with various hormones having an influence on food intake [12]. Although on a short-term basis the palatable foods can provide some relief from negative emotions and mood states, in the long term the chronic consumption of calorically-rich foods ultimately leads to overeating and consequently to obesity [8,9,10,11,12,13], which in turn promotes vulnerability to depression and anxiety [9]. Klatzkin et al. [14] determined that stress-induced negative affect predicted an increased snack intake, and that higher perceived life stress was associated with heightened emotional relief upon snacking. A systematic review and meta-analysis by Khaled et al. [15] confirmed that stress contributed to unhealthy dietary patterns (high in sweets, fat products, energy dense foods, and low in fruits, vegetables, fish, and unsaturated fats). Likewise, Gemesi et al. [16] suggested that specific comfort foods and substitutes were preferred by individuals in stressful situations. Their online survey, conducted among 1234 German participants, showed that sweets, especially chocolate and cookies, were the most preferred. Snacking may be related to the need to chew. Mastication (chewing) under stressful conditions attenuates the stress-induced increases in plasma corticosterone and catecholamines, as well as the expression of stress-related substances. Moreover, chewing reduces stress-induced changes in the central nervous system morphology [17]. All of this, combined with limiting physical activity, increases the risk of weight gain and perpetuates the vicious cycle of obesity [8]. It should be emphasized that this group of patients, due to the above-mentioned reasons and due to the greater risk of complications of COVID-19, was particularly and acutely affected by the consequences of the pandemic. Obesity, apart from chronic inflammation, is often associated with arterial hypertension, diabetes, and obstructive sleep apnea, which increase the risk of a severe course of COVID-19 [3,18,19,20,21].

Considering the above, the aim of the survey was to assess the impact of the COVID-19 pandemic on the mood and diet of patients with obesity undergoing bariatric surgery. It also seemed important to assess the patients’ eating behavior in the context of COVID-19 risk and obesity comorbidities. All of these factors may significantly affect the effectiveness of surgical treatment and are essential for organizing the care of bariatric patients during emergencies.

## 2. Materials and Methods

This cross-sectional study was carried out among 312 subjects, including patients in the process of qualifying for bariatric surgery, as well as after the surgical treatment of obesity. It was conducted by an online survey method using a proprietary questionnaire. The questionnaire consisted of 32 questions, both single (27) and multiple choice (5), as well as open (4) and closed (28) questions. The open questions were regarding body weight, age, and height. The closed questions were regarding the stage of bariatric treatment, diseases coexisting with obesity, and the psycho-dietetic reactions to the epidemiological situation. Additionally, we analyzed the relationships between psycho-dietary responses to the epidemiological situation and the occurrence of depressed mood, loss of dietary control, and weight gain in relation to the obesity comorbidities and opinion on their negative impact on the prognosis of COVID-19, as well as the stage of bariatric treatment.

The questionnaire was positively evaluated and approved by independent specialists in the field of bariatric surgery: surgeons; dieticians; and psychologists.

The Google Forms-based survey was published and distributed via social media, in cooperation with the Polish Bariatric Patients Society. All of the data were anonymized and did not include patient identifiers.

The inclusion criterion was the patient’s declaration of bariatric treatment (before and after surgery). The absence of such a declaration prevented people from answering the subsequent questions and consequently completing the questionnaire. The questionnaire was posted on the Bariatric Patients’ Association website, to minimize the risk of research misconduct, e.g., recruiting the wrong participants. This association brings together both people who are being prepared for the surgical treatment of obesity and those after bariatric surgery. In this way, the people who planned to undergo conservative treatment or were being treated in this way could not participate in the survey. The survey was conducted during the period of the greatest restrictions resulting from the COVID-19 pandemic imposed by the Polish Ministry of Health

The study was conducted according to the guidelines of the Declaration of Helsinki, and approved by the Bioethics Committee of the Medical University of Warsaw (AKBE/183/2020).

### Statistical Analysis

Statistical analysis was performed in IBM SPSS Statistics 27.0 software. We used ρ Spearman’s correlation, logistic regression analysis, and χ^2^ test of. Statistical significance was set at *p* < 0.05. The associations between the variables were analyzed in contingency tables with degrees of freedom equal to two. Assuming the statistical power to be equal to 0.80 and effect size to be equal to 0.2 in terms of Cohen’s w effect size measure, we needed a sample of at least 241 participants. The sample of 312 subjects gave us additional power and the potential to detect any effect equal to at least w = 0.18 as statistically significant.

## 3. Results

### 3.1. Characteristics of the Study Group

There were 312 subjects (M = 41.18, SD = 9.95), 265 women (84.9%) aged 19–73 years (M = 40.29, SD = 9.65) and 47 men (15.1%) aged 25–65 years (M = 105.56, SD = 26.96). The body weight of the patients ranged from 53–205 kg (M = 168.40 kg, SD = 7.64) and the BMI ranged from 18.69–82.62 kg/m^2^ (M = 37.27 kg/m^2^, SD = 9.04). More than half of the respondents (53%) had undergone bariatric surgery (23% were more than a year out from surgery), the remaining patients were at the stage of qualifying for surgery. At least one comorbid disease was diagnosed in 196 subjects (62.8%). The obesity comorbidities were mainly arterial hypertension (34.6%) and insulin resistance. The vast majority of the respondents believed that the impact of obesity and its complications on the prognosis of coronavirus infection was negative (Table 1).

### 3.2. Psycho-Dietary Reactions to the Epidemiological Situation of All Respondents

About 70% of all respondents reacted to the epidemiological situation in varying degrees: irritability (72.4%), anxiety about their own health (69.6%). More than 60% of people ate without being hungry (66%), ate regularly (of which 45.2%—frequently), felt that important life matters were out of control (63.8%), and felt the need for dietary consultation (61.2%). The consumption of alcohol (29.9%) and fatty foods (35.3%) were least frequently indicated (Table 2).

When asked about the impact of the epidemiological situation on mood, most people indicated a decrease in mood (62.8%), and 4.2% felt an improvement. The rest (33.0%) did not observe any changes. A similar percentage of all respondents felt that the epidemiological situation resulted in an increase in body weight or no change (about 40%). As far as the influence of the epidemiological situation on body weight is concerned—increase as well as lack of influence was found by a similar percentage of the respondents (about 40%). The most common reason for an increase in body weight was a decrease in physical activity (27.2%) (Table 3).

Based on ρ Spearman’s correlation coefficient, there were no statistically significant correlations between the age of the subjects and the frequency of response to the epidemiological situation. Logistic regression analysis showed no relationship between the subjects’ age and depressed mood, loss of diet control, and weight gain (*p* > 0.05).

### 3.3. Dietary Habits of All Respondents

A total of 74% of all of the subjects snacked between meals. Sweets were consumed most frequently (50.6%). This was followed by fruit (28.5%), salty snacks (25.0%), and cold cuts (20.2%). Almost half of the respondents consumed sweet drinks, mostly carbonated drinks (Table 4).

The most common reason for snacking between meals was boredom (18.6%) (Table 5).

Almost 60% of all respondents experienced a loss of control over their diet and made attempts to improve it, including several times in the case of 45% of the respondents (Table 5).

### 3.4. Obesity Comorbidities and Opinions on the Importance of Obesity and Its Complications on Prognosis in COVID-19 Responses to the Epidemiological Situation

Eating after meals (7.7% vs. 4.3%, *p* = 0.01), eating fatty meals (4.1% vs. 3.4%, *p* = 0.025), and need for psychological support (17.9% vs. 7.8%, *p* = 0.007) were significantly more prevalent among those who had comorbidities. The subjects who had no comorbidities were significantly more likely to eat regularly (56.9% vs. 38.3%, *p* = 0.005) (Table 6).

The respondents who believed that obesity and its complications had a negative impact on the prognosis of coronavirus infection had a statistically significant higher prevalence of health anxiety (22.1% vs. 5.6%, *p* = 0. 001), feeling that important life issues are out of control (22.5% vs. 15.7%, *p* = 0.001), irritability (29.4% vs. 18.5%, *p* = 0.001), need for psychological support (16.7% vs. 9.3%, *p* = 0.045), and need for dietary consultation (21.6% vs. 10.2%, *p* = 0.011) (Table 6).

Loss of control was significantly more common among those with comorbidities (77.0% vs. 62.1%; *p* = 0.005) (Table 7). Patients who believed that obesity and its complications had a negative impact on the prognosis of coronavirus infection were significantly more likely to experience depressed mood (71.1% vs. 47.2%, *p* = 0.001) than others (Table 7).

### 3.5. Stage of Bariatric Treatment and Responses to the Epidemiological Situation

Bariatric surgery patients had a statistically significant lower incidence of feeling physically hungry (40.4% vs. 26.7%, *p* = 0.020), eating after meals (65.7% vs. 41.8%, *p* = 0.001), eating fatty foods (74.1% vs. 54.1%, *p* = 0.001), and eating to the point of overeating (63.3% vs. 46.6%, *p* = 0.002) (Table 8).

The patients before surgery were significantly more likely to have loss of sense of control (80.8% vs. 63.3%, *p* = 0.001), and weight gain (65.3% vs. 38.1%, *p* = 0.001) (Table 9).

## 4. Discussion

In this study, it was shown that the COVID-19 pandemic had a significant impact on both the mood and diet of patients qualified for bariatric surgery and after surgical treatment. About 70% of all patients responded with both irritability, anxiety, and about 60% experienced a depressed mood and loss of control over their diet. At the same time, more than 70% reported snacking, especially products that provide a lot of simple carbohydrates (sweets, fruit), as well as salty snacks. This behavior was probably related to a desire to improve mood and decrease anxiety [9,10,11,12,13,14,15,16,17]. In addition, the patients ate despite not feeling hungry, ate after meals, and ate past fullness. Less than half of the patients reported an increase in body weight. In our study, the declared alcohol consumption in response to lockdown was about 30%. It was proven that mood disorders and stress may result in out-of-control eating [9]. Endogenous glucocorticoids, the effector molecules of the stress response, increase the tendency to consume high-calorie (rich in sugars and fats), palatable foods [13]. An online study by Walędziak et al. [4] involving 198 patients on a waiting list for bariatric surgery found that 82.1% of the patients felt more anxiety or fear about their health and life in regard to the COVID-19 pandemic. A questionnaire survey of 50 bariatric surgery candidates conducted in Pennsylvania at three time points: initial visit to the bariatric surgery program; prior to the onset of COVID19 restrictions; and the end of the lockdown period, showed that there was an increase in emotional eating, eating past fullness, snacking behaviors, eating sweets, and drinking liquid calories between the second and third stage [22]. In an online study of both non-bariatric and bariatric patients, one third of the participants significantly changed their eating habits. A total of 19% of the participants said that snacking substantially increased during the home lockdown, while 26% reported no changes [23]. In a study by Minsky at al. [24], conducted in Israel in a group of 279 adults treated in hospital-based obesity clinics with counseling, medications, surgery, endoscopic procedures, or any combination of these methods for weight loss, 44% of the patients reported consuming more food overall, and 40% more sweet or salty processed snacks. Durão et al. [25] demonstrated that higher levels of emotional distress during lockdown were associated with increased energy-dense micronutrient-poor food consumption among postoperative bariatric patients. Increased consumption of these products, particularly sweets, was associated with higher odds of bariatric patients not decreasing their BMI [25]. A study of 156 patients one year after bariatric surgery found that 27% of them reported depression, and 36% of them reported anxiety; the dietary habits were affected in 72% of the participants, but only 15% reported better diet planning. The intake of any type of alcoholic beverage changed in only 19% of the participants (with an increase in alcohol use in 71% of these individuals) [26]. In a study conducted among patients who had undergone bariatric surgery, 13% of the participants reported moderately-severe to severe depressive symptoms, and 8% reported severe anxiety. Moreover, 30% of the participants reported a lower diet quality, and 64% reported a worsening in at least one eating behavior. A minority of the participants (10%) reported an increase in alcohol consumption [27]. In patients who were an average of 42 months after sleeve gastrectomy, 29.2% admitted to snacking. The patients who acknowledged snacking had higher self-reported body weight gain [28]. Mood and stress disorders, and the emotional eating that often accompanies them, play a key role in the proper qualification of patients for the surgical treatment of obesity. An inability to cope with stress, manifested by compulsive eating, irregularity of meals, snacking, and excessive consumption of certain foods, can negatively affect the results of surgical treatment of obesity in terms of weight gain, postoperative complications, or risk of diet-related diseases, such as hyperlipidemia.

In a study analyzing the impact of quarantine on post-bariatric patients and self-quarantine’s relationship with weight gain, it was shown that a large percentage of the patients reported increases in their depression (44.2%), loneliness (36.2%), nervousness (54.7%), snacking (62.6%), loss of control when eating (48.2%), binge eating (19.5%), decreases in social support (23.2%), healthy food eating (45.5%), and activity (55.2%) [21].

In our study, nearly 40% reported an increase in weight due to the epidemiological situation. A similar percentage reported no change. The respondents mainly indicated changes in physical activity as the cause. In the study by Walędziak et al. [4], about 50% of the respondents reported both an increase in body weight and no change/decrease in body weight. The majority of patients with weight gain declared a decrease in physical activity [4]. In the study by Naran et al. [22], the physical activity of the candidates preparing for surgery increased between the first stages of the study, but decreased again by 60% throughout the lockdown. Minsky at al. [24] showed that the patients who increased physical activity had less weight gain during lockdown. The main finding of the cross-sectional study of de Luiz et colleagues [28] was the fact that the lockdown produced a significant increase in self-reported weight gain in a sample of 48 patients with sleeve gastrectomy. This increase in body weight was associated with a decrease in physical activity and the loss of face-to-face visits to the Nutrition Unit. In the study, of 937 post-bariatric surgery patients, 67% decreased physical activity, resulting in weight gain [27]. In another study of 188 patients undergoing bariatric surgery, 83.5% reported more sedentary behaviors [26]. Athanasiadis and colleagues [21] showed a significant effect of quarantine on the reduction in body weight and physical activity of patients. Exercise during the pandemic was found to be negatively affected, with 51.8% of the respondents being less active, and 55.2% reporting a reduction in their aerobic exercise. A retrospective case-control study, conducted in Belgium in a group of 49 bariatric surgery patients operated on before and during the COVID-19 pandemic, showed that there were statistically significant differences in terms of percentage of excessive weight loss (%EWL). The %EWL was lower in the COVID-19 period compared to that in the non-COVID-19 period (82.4% vs. 91.7%). During the first postoperative year, the BMI was statistically significantly higher in the patients of the COVID-19 period after one year of follow-up. These results are linked by the authors to an increased number of meals, increased snack and alcohol consumption, a reduction in physical activity, a negative emotional state, increasing vulnerability, and symptoms of anxiety, loneliness, and depression [29].

In our study, we showed that individuals with complications of obesity were more likely to eat improperly (including eating after meals, eating fatty foods) and to lose control of their eating patterns. In contrast, those who were aware that obesity and its complications were making COVID-19 worse were more likely to be anxious about their health, feel a loss of control over important matters, and experience irritability and mood swings. Many of the studies have shown that obesity and its complications worsen COVID-19, increase the risk of severe complications, and even mortality. That is associated with increased intra-abdominal fat, endocrine and metabolic dysfunction, and systemic inflammation in the course of obesity [18,20,30]. Only 35% of our patients were aware of this risk. In the study by Walędziak et al. [4], 32% of the patients were not aware that obesity was an important risk factor for COVID-19 severity. In both cases, they were more likely to need psychological counseling, in the latter case additionally, and also dietary counseling. These results indicate that consultations with a nutritionist and psychologist can be helpful in making the patient aware of the risks arising from the disease, and can also be a support in coping with difficulties.

We found that patients being prepared for bariatric surgery compared to those undergoing surgery were significantly more likely to eat despite not being hungry, overeat, consume fatty foods, and eat until they felt full. These subjects were also more likely to experience a loss of control over their diet and weight gain. This result is supported by Jimenez et al. [23]. The non-BS participants reported a greater impact of lockdown on mood, experienced more negative changes in dietary habits, and had a higher likelihood for weight gain. This may indicate the importance of surgical treatment on changing eating habits and controlling hunger. In the latter case, the hormonal regulation of appetite and changes in taste due to bariatric surgery may play a large role [31]. Interestingly, the patients >2 years after surgery responded similarly to the non-bariatric surgery patients. Bariatric surgery status within the two previous years emerged as a protective factor for weight gain during lockdown [23]. Eating disorders, including binge-eating disorder and emotional eating, are quite common among bariatric surgery candidates. While it is well known that the eating disorders, such as binge eating and emotional eating, tend to improve, the longer-term data are inconclusive. This implies the need for regular follow-up. Unfortunately, the pandemic and associated lockdown has made this much more difficult [3,23]. For bariatric patients at further risk for obesity comorbidities and nutritional deficiencies, this support is essential. Yeo and colleagues suggest telemedicine consultations in this situation, including both telephone and online platforms. Online social support groups can also be created. It was shown that regular post-operative telephone calls addressing behavior change strategies for diet, physical activity, and nutrition can potentially decrease the incidence of weight regain [3]. The study, conducted in Israel, assessed the association between changes in the dietary and lifestyle habits and body weight, and the benefits of receiving weight management care remotely through telemedicine during lockdown. Compared to the patients not receiving multidisciplinary obesity care via telemedicine, the patients receiving this care were more likely to lose weight and also to increase participation in exercise [24]. An online survey conducted by Walędziak et al. [4] showed that 62.8% of the patients had an opportunity to contact a bariatric surgeon, 45.9%—a dietician, and 37.8%—a psychologist. At the same time, 71.3% of them benefited from online support. A study involving 396 candidates for surgical treatment of obesity compared the effect of face-to-face screening with tele-screening. It was shown that the multidisciplinary team’s decision was not significantly different between these two types of consultation [32]. Fakharian et al. emphasized that virtual consultations and telemedicine may be suitable to continue weight loss programs [20]. Minsky et al. [24] emphasized that, compared to patients not receiving obesity care via telemedicine, the patients receiving this care were more likely to lose weight and increase their participation in exercise.

## 5. Conclusions

The COVID-19 pandemic has been shown to adversely affect bariatric patients’ well-being and diet, which can exert an impact on their health, and worsen the prognosis of infection. About 70 % of all patients responded indicating they experienced irritability and anxiety, and about 60 % experienced a depressed mood and loss of control over their diet. In addition, the patients ate despite not feeling hungry, ate after meals, and ate beyond satiety. It was shown that mood disorders and stress can lead to uncontrolled eating. The respondents after bariatric surgery had better eating habits, so this form of treatment may help prevent obesity and severe coronavirus infection in patients with morbid obesity. The results of our study may be particularly useful in qualifying the patients for bariatric surgery. This should include both psychological (risk of mood disorders, eating disorders) and dietary (preferred foods, excessive consumption in stressful situations, meal regularity, compulsive eating) assessments. In addition, our results may be useful for assessing patients’ diets for diet-related diseases in the postoperative period.

The COVID-19 pandemic is a sign that such epidemiological events may be recurring and that the role of multispecialty support for bariatric patients is important, especially since it has been shown that the patients are often unaware of the health risks associated with obesity, and that those who have had bariatric surgery have had better nutritional habits

### Limitations of the Study

The most important limitations of the survey were the limited controllability of the survey (it was conducted via the Internet), the subjectivity of the patients’ responses, and the risk of unreliable responses. However, direct control of the survey, due to the ongoing epidemic, was impossible. To minimize the risk of false results, we published the questionnaire through the Bariatric Patient Association. It would be advisable to conduct the study in a larger group of patients.

Further research is needed on the relationship between mood, stress, and emotional eating, as well as the preference/consumption of specific foods and eating in stressful situations. This is particularly important for bariatric patients, as it can significantly affect the effectiveness of the surgery in terms of weight loss, as well as the postoperative complications.

## Figures and Tables

**Table 1:** Characteristics of the study group by stage of bariatric treatment, obesity comorbidities, and opinions on the effect of obesity on the prognosis of COVID-19 (*n* = 312).

Stage of Bariatric Treatment	*n*	*%*
Preparing for bariatric surgery	146	46.8
1–3 months after bariatric surgery	24	7.7
4–6 months after bariatric surgery	29	9.3
6–12 months after bariatric surgery	41	13.1
>12 months after bariatric surgery	72	23.1
Total	312	100
Obesity comorbidities	*n*	%
Type 2 diabetes	35	11.2
Insulin resistance	69	22.1
Hypertension	108	34.6
Cardiovascular diseases	25	8.0
Hyperlipidemia	33	10.6
Sleep apnea syndrome	21	6.7
Depression	45	14.4
Anxiety Disorders	16	5.1
Other mental illnesses	5	1.6
Total	196	62.8
The negative impact of obesity and its complications on the prognosis of COVID-19	*n*	%
No	20	6.4
Don’t know	88	28.2
Yes	204	65.4
Total	312	100

*n*—number of responses, % of the sample.

**Table 2:** Psycho-dietary responses to the epidemiological situation (*n* = 312).

		Frequency		
Responses to the Epidemiological Situation		No	Yes, Sometimes	Yes, Often
Anxiety about one’s own health	*n*	95	166	51
	%	30.4	53.2	16.3
Feeling that important things in life are out of control	*n*	113	136	63
	%	36.2	43.6	20.2
Irritability	*n*	86	146	80
	%	27.6	46.8	25.6
Eating without hungry	*n*	106	131	75
	%	34.0	42.0	24.0
Eating regularly	*n*	108	63	141
	%	34.6	20.2	45.2
Eating after meals	*n*	170	122	20
	%	54.5	39.1	6.4
Eating fatty foods	*n*	202	98	12
	%	64.7	31.4	3.8
Alcohol drinking	*n*	221	70	21
	%	70.8	22.4	6.7
Overeating	*n*	173	119	20
	%	55.4	38.1	6.4
Need for psychological counseling	*n*	154	114	44
	%	49.4	36.5	14.1
Need for dietary counseling	*n*	121	136	55
	%	38.8	43.6	17.6

*n*—number of responses, % of the sample; results do not add up to 100% because more than one answer could be selected.

**Table 3:** Effect of the epidemiological situation on the body weight of subjects and the reasons for the increase in body weight (*n* = 312).

Effect on Body Weight	*n*	%
Weight gain	121	38.8
No change	126	40.4
Weight loss	65	20.8
Total	312	100
Causes of weight change	*n*	%
Stress and anxiety resulting from the situation	38	12.2
Change in physical activity	85	27.2
Change in current diet	42	13.5
I don’t know	21	6.7
Total	186	59.6

*n*—number of responses, % of the sample.

**Table 4:** Foods and beverages snacked between meals by all respondents (*n* = 312).

Food Products	*n*	%
Sweets (cookies, cakes, candies, chocolates, ice cream)	158	50.6
Salty snacks (sticks, crisps)	78	25.0
Vegetables	38	12.2
Fruit	89	28.5
Meat products, e.g., sausages	63	20.2
Cheeses	56	17.9
Sweet dairy products (yoghurts, cheese, etc.)	50	16.0
Bread	45	14.4
I don’t snack	81	26.0
Sweet drinks	*n*	%
Sweet carbonated drinks such as Cola, Fanta, Sprite	48	15.4
Energy drinks	14	4.5
Juices	33	10.6
Sweetened coffee	23	7.4
Sweetened tea	27	8.7
I don’t consume sweet drinks	167	53.5

*n*—number of responses, % of the sample; results do not add up to 100% because more than one answer could be selected.

**Table 5:** Reasons for snacking between meals and the negative consequences of the lack of control over the current diet and the attempts to correct dietary indiscretion by all respondents.

Reasons for Snacking	*n*	%
Boredom	58	18.6
The need to please myself, “because I like it”	49	15.6
Stress	52	16.7
Need to calm down	38	12.2
I eat while doing other activities, e.g., talking on the phone, watching TV, reading a book	34	10.9
I don’t snack	81	26.0
Negative consequences of not having control over your diet (suffering remorse and problems, e.g., health, financial)	*n*	%
No	44	14.1
Yes, sometimes	101	32.4
Yes, often	78	25.0
Loss of control	89	28.5
Total	312	100
Making attempts to correct dietary errors	*n*	%
No/ No such problem	129	41.3
Yes, several times	141	45.2
Yes, repeatedly	42	13.5
Total	312	100.0

*n*—number of responses, % of the sample; results do not add up to 100% because more than one answer could be selected.

**Table 6:** Psycho-dietetic reactions to the epidemiological situation depending on obesity comorbidities and opinions on their negative impact on the prognosis of COVID-19.

		Obesity Comorbidities							Negative Obesity and Its Comorbidities Impact on the Prognosis of COVID-19						
		No (*n* = 116)		Yes (*n* = 196)					No/I Don’t Know (*n* = 108)		Yes (*n* = 204)				
Psycho-Dietetic Reactions	Frequency	*n*	%	*n*	%	χ^2^	*df*	*p*	*n*	%	*n*	%	χ^2^	*df*	*p*
Anxiety about one’s own health	Never	39	33.6	56	28.6	4.96	2	0.084	52	48.1	43	21.1	30.24	2	0.001
	Sometimes	65	56.0	101	51.5				50	46.3	116	56.9			
	Often	12	10.3	39	19.9				6	5.6	45	22.1			
Feeling that important things in life are out of control	Never	48	41.4	65	33.2	2.77	2	0.251	58	53.7	55	27.0	22.00	2	0.001
	Sometimes	49	42.2	87	44.4				33	30.6	103	50.5			
	Often	19	16.4	44	22.4				17	15.7	46	22.5			
Irritability	Never	31	26.7	55	28.1	0.86	2	0.652	46	42.6	40	19.6	19.01	2	0.001
	Sometimes	58	50.0	88	44.9				42	38.9	104	51.0			
	Often	27	23.3	53	27.0				20	18.5	60	29.4			
Eating without hungry	Never	43	37.1	63	32.1	5.94	2	0.051	44	40.7	62	30.4	5.09	2	0.079
	Sometimes	54	46.6	77	39.3				45	41.7	86	42.2			
	Often	19	16.4	56	28.6				19	17.6	56	27.5			
Eating regularly	Never	30	25.9	78	39.8	10.48	2	0.005	35	32.4	73	35.8	1.63	2	0.443
	Sometimes	20	17.2	43	21.9				19	17.6	44	21.6			
	Often	66	56.9	75	38.3				54	50.0	87	42.6			
Eating after meals	Never	76	65.5	94	48.0	9.16	2	0.010	63	58.3	107	52.5	1.45	2	0.485
	Sometimes	35	30.2	87	44.4				40	37.0	82	40.2			
	Often	5	4.3	15	7.7				5	4.6	15	7.4			
Eating fatty foods	Never	86	74.1	116	59.2	7.35	2	0.025	70	64.8	132	64.7	0.54	2	0.762
	Sometimes	26	22.4	72	36.7				35	32.4	63	30.9			
	Often	4	3.4	8	4.1				3	2.8	9	4.4			
Alcohol drinking	Never	82	70.7	139	70.9	0.01	2	0.996	75	69.4	146	71.6	0.87	2	0.648
	Sometimes	26	22.4	44	22.4				27	25.0	43	21.1			
	Often	8	6.9	13	6.6				6	5.6	15	7.4			
Overeating	Never	73	62.9	100	51.0	4.35	2	0.114	66	61.1	107	52.5	2.38	2	0.304
	Sometimes	36	31.0	83	42.3				35	32.4	84	41.2			
	Often	7	6.0	13	6.6				7	6.5	13	6.4			
Need for psychological counseling	Never	69	59.5	85	43.4	9.83	2	0.007	63	58.3	91	44.6	6.21	2	0.045
	Sometimes	38	32.8	76	38.8				35	32.4	79	38.7			
	Often	9	7.8	35	17.9				10	9.3	34	16.7			
Need for dietary counseling	Never	47	40.5	74	37.8	4.00	2	0.135	52	48.1	69	33.8	9.07	2	0.011
	Sometimes	55	47.4	81	41.3				45	41.7	91	44.6			
	Often	14	12.1	41	20.9				11	10.2	44	21.6			

*n*—number of responses, % of the sample; χ^2^—value of the independence test; *df*—the number of degrees of freedom; *p*—statistical significance.

**Table 7:** The influence of obesity comorbidities and opinions on their negative impact on the prognosis of COVID-19 on the occurrence of depressed mood loss of diet control, and weight gain.

	Obesity Comorbidities							Negative Obesity and Its Comorbidities Impact on the Prognosis of COVID-19						
	No		Yes					No/I don’t know		Yes				
	*n*	%	*n*	%	χ^2^	*df*	*p*	*n*	%	*n*	%	χ^2^	*df*	*p*
Depressed mood (*n* = 196)	65	56.0	131	66.8	3.64	1	0.056	51	47.2	145	71.1	17.21	1	0.001
Loss of diet control (*n* = 223)	72	62.1	151	77.0	8.01	1	0.005	70	64.8	153	75.0	3.59	1	0.058
Weight gain (*n* = 117)	32	44.4	85	56.3	2.74	1	0.098	31	44.3	86	56.2	2.74	1	0.098

*n*—number of responses, % of the sample; χ^2^—value of the independence test; *df*—the number of degrees of freedom; *p*—statistical significance.

**Table 8:** Psycho-dietetic reactions to the epidemiological situation depending on stage of bariatric treatment.

		Stage of Bariatric Treatment						
		Before Surgery (*n* = 146)		After Surgery (*n* = 166)				
Psycho-Dietetic Reactions	Frequency	*n*	%	*n*	%	χ^2^	*df*	*p*
Anxiety about one’s own health	Never	44	30.1	51	30.7	2.62	2	0.271
	Sometimes	73	50.0	93	56.0			
	Often	29	19.9	22	13.3			
Feeling that important things in life are out of control	Never	48	32.9	65	39.2			
	Sometimes	65	44.5	71	42.8	1.69	2	0.43
	Often	33	22.6	30	18.1			
Irritability	Never	38	26.0	48	28.9	3.34	2	0.188
	Sometimes	76	52.1	70	42.2			
	Often	32	21.9	48	28.9			
Eating without hungry	Never	39	26.7	67	40.4	7.83	2	0.020
	Sometimes	64	43.8	67	40.4			
	Often	43	29.5	32	19.3			
Eating regularly	Never	59	40.4	49	29.5	5.65	2	0.059
	Sometimes	31	21.2	32	19.3			
	Often	56	38.4	85	51.2			
Eating after meals	Never	61	41.8	109	65.7	17.87	2	0.001
	Sometimes	73	50.0	49	29.5			
	Often	12	8.2	8	4.8			
Eating fatty foods	Never	79	54.1	123	74.1	14.67	2	0.001
	Sometimes	58	39.7	40	24.1			
	Often	9	6.2	3	1.8			
Alcohol drinking	Never	107	73.3	114	68.7	1.80	2	0.408
	Sometimes	32	21.9	38	22.9			
	Often	7	4.8	14	8.4			
Overeating	Never	68	46.6	105	63.3	12.09	2	0.002
	Sometimes	63	43.2	56	33.7			
	Often	15	10.3	5	3.0			
Need for psychological counseling	Never	69	47.3	85	51.2	1.98	2	0.371
	Sometimes	59	40.4	55	33.1			
	Often	18	12.3	26	15.7			
Need for dietary counseling	Never	48	32.9	73	44.0	4.36	2	0.113
	Sometimes	68	46.6	68	41.0			
	Often	30	20.5	25	15.1			

*n*—number of responses, % of the sample; χ^2^—value of the independence test; *df*—the number of degrees of freedom; *p*—statistical significance.

**Table 9:** The influence of the stage of bariatric treatment on the occurrence of depressed mood loss of diet control, and weight gain.

	Stage of Bariatric Treatment						
	Before Surgery		After Surgery				
	*n*	%	*n*	%	χ^2^	*df*	*p*
Depressed mood (*n* = 196)	93	63.7	103	62.0	0.09	1	0.763
Loss of diet control (*n* = 223)	118	80.8	105	63.3	11.76	1	0.001
Weight gain (*n* = 117)	77	65.3	40	38.1	16.43	1	0.001

*n*—number of responses, % of the sample; χ^2^—value of the independence test; *df*—the number of degrees of freedom; *p*—statistical significance.

## Data Availability

The data presented in this study are available upon request from the corresponding author. The data are not publicly available as they are still being used for analysis and manuscript writing.
